# Demographic Differences in Falls Prevention Outcomes: Insights From Evidence-Based Falls Prevention Programs

**DOI:** 10.1093/geroni/igaf122.1754

**Published:** 2025-12-31

**Authors:** Uma Kelekar, Mahederemariam Dagne, Sara Pappa, Catherine Diaz-Asper, J Taylor Harden, Shelly Aboagye, Erin Staker, Patricia Heyn

**Affiliations:** Marymount University, Arlington, Virginia, United States; Marymount University, Arlington, Virginia, United States; Marymount University, Arlington, Virginia, United States; Marymount University, Arlington, Virginia, United States; Marymount University, Fairfax Station, Virginia, United States; Marymount University, Arlington, Virginia, United States; Center for Optimal Aging, Arlington, Virginia, United States; Marymount University, Arlington, Virginia, United States

## Abstract

Falls remain a leading cause of injury among older adults in the United States, with prevalent disparities influenced by demographic factors. Race/ethnicity was reported to be one factor influencing overall falls risk. The Administration for Community Living (ACL) launched nationwide Evidence-Based Falls Prevention Programs (EBFPPs) to counter falls among older adults. This provided a unique opportunity to analyze program effectiveness across the U.S. among racial and ethnic minorities. For this study, we utilize the results from the large-scale EBFPP datasets to assess variations in falls prevention outcomes related to race/ethnicity. The participant data (N = 203,838) is representative of various racial and ethnic groups, with a majority of participants identifying as NH White (79.9%), followed by NH Black (9.03%), Hispanic (5.12%), and other races or ethnicities. Across all participants, total falls declined by 20%, fear of falls declined by 30%, and injurious falls declined by 10%. There were statistically significant differences amongst the racial-ethnic groups in all metrics analyzed, with NH white and Hispanic participants consistently reporting the highest reductions across all falls metrics while NH Black participants reported lower reduction rates. This work provides valuable insights for researchers, policymakers, public health officials, and aging service providers to optimize falls prevention interventions to better serve diverse and high-risk populations indicating the need for incorporating targeted outreach efforts, culturally competent program modifications, and state-level policy considerations to enhance equity in falls prevention.

